# Introductions of West Nile Virus Strains to Mexico

**DOI:** 10.3201/eid1202.050871

**Published:** 2006-02

**Authors:** Eleanor Deardorff, José G. Estrada-Franco, Aaron C. Brault, Roberto Navarro-Lopez, Arturo Campomanes-Cortes, Pedro Paz-Ramirez, Mario Solis-Hernandez, Wanichaya N. Ramey, C. Todd Davis, David W.C. Beasley, Robert B. Tesh, Alan D.T. Barrett, Scott C. Weaver

**Affiliations:** * University of Texas Medical Branch, Galveston, Texas, USA;; † University of California, Davis, California, USA;; ‡ Comisión México-Estados Unidos para la Prevención de la Fiebre Aftosa y Otras Enfermedades Exóticas de los Animales, Mexico City, Mexico

**Keywords:** West Nile virus, arbovirus, flavivirus, introduction, equine, avian, phylogenetics, dispatch

## Abstract

Complete genome sequencing of 22 West Nile virus isolates suggested 2 independent introductions into Mexico. A previously identified mouse-attenuated glycosylation variant was introduced into southern Mexico through the southeastern United States, while a common US genotype appears to have been introduced incrementally into northern Mexico through the southwestern United States.

West Nile virus (WNV), a mosquitoborne flavivirus for which birds serve as reservoir and amplification hosts, was introduced into New York in 1999 (*1*) and spread across the United States to California by 2003 (*2*). By 2002, serosurveys demonstrated WNV circulation in >6 eastern Mexican states and along its northern border with the United States (*3**–**5*). This pattern of WNV appearance in Mexico suggested a southwesterly spread across the United States and into northeastern Mexico through Texas. However, in the spring of 2003, the first WNV isolate found in Mexico was obtained from a raven in the southeastern state of Tabasco (*3*). If WNV reached southern Mexico by incremental spread through northern Mexico from Texas, the index isolate would have been expected sooner and in a northern Mexican state. Phylogenetic analyses showed the raven isolate to be unexpectedly divergent from contemporary Texas strains, but exact relationships and a route of entry could not be determined by using premembrane and envelope glycoprotein (prM-E) sequences (*3*).

The divergence between the southern Mexican raven and Texas isolates suggested that WNV arrived in southern Mexico by an alternate route, perhaps the Caribbean. After its spread throughout the northeastern United States, WNV appeared abruptly in Florida in 2001, appearing to bypass several mid-Atlantic states. This pattern could be explained by spread of migratory birds (*6*); the Atlantic coast flyway overlaps both New York and Florida, while the Mississippi Valley flyway overlaps both Louisiana and the Yucatan Peninsula of Mexico. Longitudinal avian serosurveys that began in 2000 showed WNV seropositivity in at least 3 migratory and 2 resident bird species captured in the Yucatan Peninsula from 2002 to 2003 (*7*). Thus, migratory birds may have carried WNV from the southeastern United States into Mexico, either directly or through the Caribbean. Serosurveys have suggested WNV circulation among birds in various Caribbean islands since 2002 (*8**–**10*).

The possibility of a third WNV introduction into Mexico at the California border must also be considered. A 2003 horse isolate from the northern Mexican state of Nuevo Leon was closely related to Texas isolates from 2002 (*11*), based on its prM-E sequence. We do not know whether WNV reached California from Texas and the Midwest by crossing the Rocky Mountains or by traveling first into northern Mexico and subsequently spreading north from Baja California. The latter route is suggested by the geographic link with the first detection of WNV in southeastern California (*2*).

The reported incidence of human West Nile encephalitis is much greater on the US (California) than on the Mexican (Baja California and Sonora) side of the common border. Possible explanations for this discrepancy include differences in disease surveillance and reporting. Another possibility is that the WNV strains circulating in Mexico are attenuated compared to US strains, and the identification of a murine-attenuated glycosylation variant in Tabasco State (*12*) is consistent with this hypothesis.

## The Study

**Figure 1:**
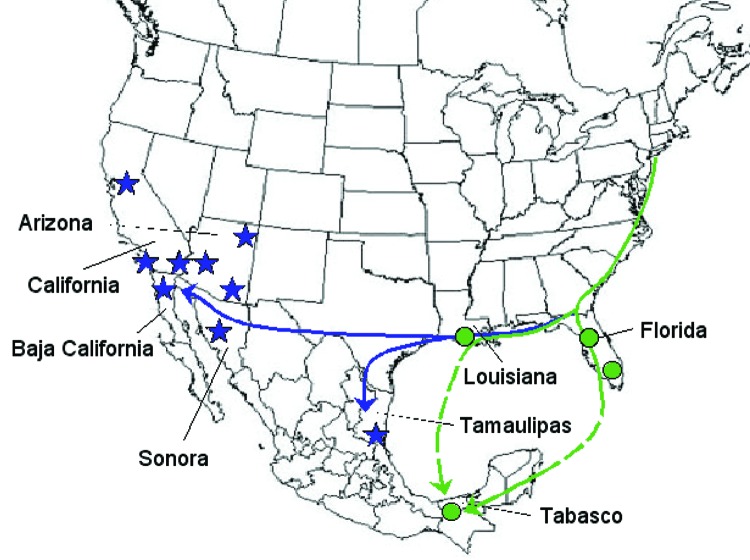
Map showing hypothetical routes of West Nile virus introduction into Mexico. Circles indicate locations of isolates in the Florida-Louisiana-Tabasco 2001–2003 clade (Figure 2). Stars indicate locations of isolates in the California-Arizona-northern Mexico clade.

To investigate possible routes of WNV entry into Mexico from the United States, 9 isolates from Mexico (all strains available) and 13 strains isolated in the United States from hypothetical points of introduction into Mexico (2 from Florida, 2 from Louisiana, 3 from Arizona, and 6 from California) were compared. Isolates from several northern Mexican states, 1 from Sonora, 1 from Tamaulipas, and 7 from Baja California, were obtained from a variety of birds and from a horse (Table, Figure 1) by injection of Vero cells. RNA was extracted from first or second Vero cell passages by using the QIAamp Viral RNA Mini-kit (Qiagen Inc, Valencia, CA, USA). Reverse transcription–polymerase chain reactions (RT-PCRs) were performed to amplify the complete WNV genome in 6 overlapping amplicons by using primers described previously (12). Amplicons were purified from agarose gels by using the QIAquick gel-extraction kit (Qiagen), and both strands were sequenced directly by using the PCR primers and the BigDye Terminator v3.1 Cycle Sequencing Kit (Applied BioSystems, Foster City, CA, USA) with a 3100 Genetic Analyzer (Applied Biosystems).

**Table:** West Nile virus isolates included in the phylogenetic analyses*

GenBank No.	Strain	Year	Host	Location†
AB185914	(NY)I	1999	Not reported	New York
AB185915	(NY)II	1999	Not reported	New York
AB185916	(NY)III	1999	Not reported	New York
AB185917	(NY)IV	1999	Not reported	New York
AF196835	NY '99	1999	Flamingo	New York
AF202541	NY	1999	Human	New York
AF206518	2741	1999	Connecticut	
AF260967	NY99-eqhs	1999	Equine	New York
AF260968	Eg101	1951	Human	Egypt
AF260969	RO97-50	1996	Romania	
AF317203	VLG-4	1999	Human	Russia
AF404753	crow265	2000	Crow	Maryland
AF404754	MQ5488	2000	New Jersey	
AF404755	grouse3282	2000	Grouse	New York
AF404756	crow3356	2000	Crow	New York
AF404757	Italy equine	1998	Equine	Italy
AF481864	IS-98 STD	1998	Store	Israel
AF533540	US Hum. 1	2001	Human	New York
AY185911	V1151	2002	Mosquito	Texas
AY262283	Kenya3829	1998	Mosquito	Kenya
AY268132	PaAn001	2000	Equine	France
AY268133	PaH001	1997	Human	Tunisia
AY277252	27889	2003	Human	Russia
AY278441	Ast99-901	1999	Human	Russia
AY278442	Vlg00 27924	2000	Human	Russia
AY289214	TVP 8533	2002	Human	Beaumont, Texas
AY490240	Chin-01	2003	Not reported	China
AY660002	TM171-03	2003	Raven	Tabasco
AY701412	96-111	1996	Equine	Morocco
AY701413	04.05	2003	Equine	Morocco
AY712945	Bird 1153	2003	Bird	Harris Co., Texas
AY712946	Bird 1171	2003	Bird	Harris Co., Texas
AY712947	Bird 1461	2003	Bird	Harris Co., Texas
AY712948	v4369	2003	Mosquito	Harris Co., Texas
AY842931	385-99	1999	Not reported	New York
D00246	Kunjin MRM61C	1960	*Culex* spp.	Australia
M12294	Uganda WNFCG	1937	Human	Uganda
**DQ080070**	**TVP 9115**	**2003**	**Grackle**	**Sonora, Mexico**
**DQ080069**	**TVP 9117**	**2003**	**Horse**	**Tamaulipas**
**DQ080068**	**TVP 9218**	**2003**	**Blue heron**	**Baja California, Mexico**
**DQ080067**	**TVP 9219**	**2003**	**Green heron**	**Baja California, Mexico**
**DQ080066**	**TVP 9220**	**2003**	**Cormorant**	**Baja California, Mexico**
**DQ080065**	**TVP 9221**	**2003**	**Grackle**	**Baja California, Mexico**
**DQ080064**	**TVP 9222**	**2003**	**Coot**	**Baja California, Mexico**
**DQ080063**	**TVP 9223**	**2003**	**Pigeon**	**Baja California, Mexico**
**DQ080060**	**Cc**	**2004**	**Raven**	**Baja California, Mexico**
**DQ080072**	**FL232**	**2001**	**Catbird**	**Palm Beach Co., Florida**
**DQ080071**	**FL234**	**2002**	**Equine**	**Sumter Co., Florida**
**DQ080062**	**TWN 165**	**2002**	**Mosquito**	**Iberia Co., Louisiana**
**DQ080061**	**TWN 496**	**2004**	**Northern cardinal**	**Iberia Co., Louisiana**
**DQ080051**	**AZ-03-1623**	**2003**	**Cohise Co., Arizona**	
**DQ080052**	**Az-03-1681**	**2003**	***Cx. tarsalis***	**Maricopoa Co., Arizona**
**DQ080053**	**Az-03-1799**	**2003**	***Cx. tarsalis***	**Apache Co., Arizona**
**DQ080054**	**CA-03 GRLA**	**2003**	***Cx. quinquefasciatus***	**Los Angeles, California**
**DQ080055**	**CA-03 IMPR 102**	**2003**	**Imperial Valley, California**	
**DQ080056**	**CA-03 IMPR 1075**	**2003**	***Cx. tarsalis***	**Imperial Valley, California**
**DQ080057**	**CA-03S0333081**	**2003**	**Crow**	**Arcadia, California**
**DQ080058**	**CA-03S0334814**	**2003**	**Crow**	**Arcadia, California**
**DQ080059**	**CA-04 04-7168**	**2003**	**Yellow-billed magpie**	**Sacramento, California**

*Newly sequenced strains are printed in **bold text**. †Locality and state (Mexico and United States) or country of isolation.

Complete genomic sequences excluding the 5´ and 3´ terminal 20 nucleotides (representing primers incorporated into amplicons) were aligned with all homologous WNV sequences from the GenBank library by using ClustalW. Sequences were analyzed by using maximum parsimony and neighbor-joining programs implemented in the PAUP 4.0 software package (*13*) as well as Bayesian analysis using MRBAYES v3.0 (*14*) with 100,000 generations, a general time-reversible model with empirically estimated base frequencies, and either a codon position-specific (for the open reading frame alone) or a gamma distribution of substitution rates among nucleotide sites.

**Figure 2:**
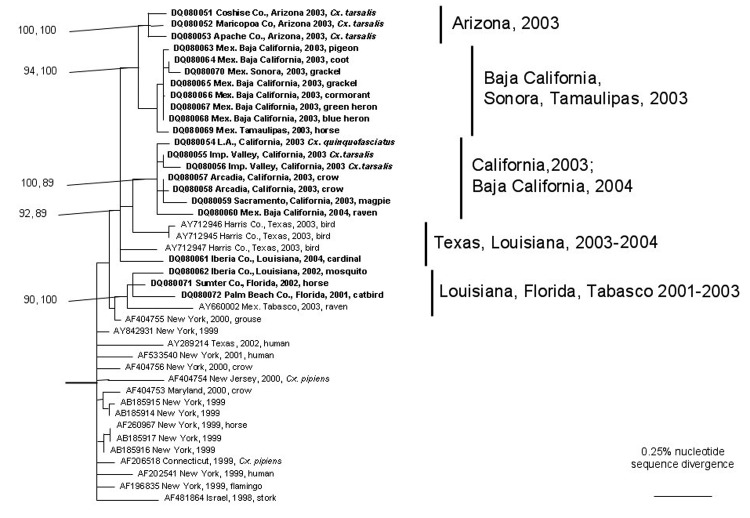
Phylogenetic tree generated from the complete open reading frame of West Nile virus sequences using a Bayesian analysis. Virus strains are labeled by GenBank accession number followed by the state and/or country, year, and host of isolation. Numbers indicate Bayesian probability values followed by neighbor-joining bootstrap values for groups to the right. The tree was rooted by using an outgroup comprised of Old World strains of West Nile virus, including a lineage 2 strain (Table).

All phylogenetic trees placed the North American WNV isolates into monophyletic groups with strong bootstrap and Bayesian support values; the tree generated using the Bayesian analyses is presented in Figure 2. The Tabasco 2003 isolate grouped with 3 strains from 2001 and 2002 in Florida and Louisiana and more distantly with a New York isolate from 2000, with strong Bayesian probability and bootstrap support; inclusion of the New York grouse strain was weakly supported (bootstrap and Bayesian probability values <80%). In contrast, a 2004 Louisiana isolate and other recent strains from Texas were positioned basally to the large clade containing all northern Mexico, California, and Arizona isolates. This California/Arizona/northern Mexico group was highly conserved, with <0.5% nucleotide and 0.04% amino acid sequence divergence. The 2003 Tabasco strain was phylogenetically distinct from all other Mexico isolates, which grouped with the California and Arizona isolates. Surprisingly, despite the greater geographic distances between Tamaulipas and Baja California/Sonora, compared to the distance between Tamaulipas and Texas, the Tamaulipas WNV strains grouped more closely with strains from Baja California and Sonora than with those from Texas.

Compared to the Tabasco strain, the other Mexican isolates differed by 0.55%–0.66% nucleotide sequence divergence across the genome. The gene with the most sequence divergence was prM, with 0.72%–1.4% divergence from the Tabasco strain. However, the 5´ untranslated region was more variable with 3.0%–4.6% divergence. The most conserved gene was NS2B, with 0.0%–0.24% divergence from the Tabasco strain. The E gene, often used for phylogenetic analyses, had 0.46%–0.66% sequence divergence.

Of the Mexican WNV isolates, only the 2003 Tabasco raven isolate had the E-156 Pro residue, which ablates the N-linked glycosylation site found in most North American strains. In addition, 2 other WNV isolates (GenBank accession nos. AY490240 and AF260968) share this E-156 Pro residue despite their geographic diversity (China and Egypt, respectively) and their placement in different lineages. Although the paraphyletic nature of this Pro mutation suggests that it could be selected either during laboratory isolation or passage, its identification in the low-passage Tabasco isolate may indicate its presence in nature.

## Conclusions

Our data support the hypothesis that WNV entered Mexico through at least 2 independent introductions. The introduction detected by the first virus isolation in May 2003 from a raven in Tabasco State probably occurred from a migratory bird that flew southward from the southeastern United States to the Gulf of Mexico or the Caribbean and bypassed northern Mexico. The isolation and sequencing of WNV isolates from islands in the Caribbean may shed further light on how WNV reached southern Mexico. However, the extreme genetic conservation of North American WNV strains may preclude identifying the exact routes of introduction. Independently, other WNV strains probably spread incrementally from the southwestern United States into northern Mexico. Both northward and southward movements of WNV between northern Mexico and California or Arizona may also occur.

Available WNV strains from Mexico indicate that the murine-attenuated, E-156 glycosylation-negative variant identified in Tabasco state may be limited in its distribution to southern Mexico, while the glycosylated variant typical of US strains is widespread in northern Mexico. However, our sampling was limited and may also be biased because many WNV isolates were from sick or dying animals; the attenuated E-156 Pro residue phenotype could be undersampled because relatively benign infections are rarely identified.

The epidemiology of WNV-associated disease in Mexico is puzzling. According to the Centers for Disease Control and Prevention, 2,470 human cases of WNV infection were confirmed during 2004 in the United States, with >80% of these from areas of California and Arizona bordering the northern Mexico states of Baja California and Sonora where many of our viral isolations were made. In contrast, only 7 human cases of WNV have been confirmed in Mexico. The cases occurred in the border states of Chihuahua (n = 4), Sonora (n = 1), and Nuevo Leon (n = 1) in 2003, and Sonora (n = 1) in 2004 (*15*). Our results of extremely low sequence divergence between the southwestern United States and northern Mexican WNV isolates indicate that this epidemiologic discrepancy is unlikely to be explained by genetic and phenotypic differences among WNV strains. The possibility that WNV circulating in Mexico has an attenuated phenotype was suggested by the murine-attenuating mutation in the Tabasco raven isolate (*12*). However, none of our northern Mexico isolates have the E-156-P attenuating mutation, and all appear extremely closely related to isolates made in southwestern areas of the United States with a high disease incidence.

Another possible explanation for the low incidence of WNV disease in Mexico is resistance in the Mexican human population, possibly because cross-protective immunity from other flavivirus infections such as dengue and St. Louis encephalitis viruses. Although St. Louis encephalitis virus is common in some areas of the continental United States, including California, dengue virus infections are rare; only 157 cases of dengue were reported in the northern states of Mexico in 2004: 25 in Sonora; 21 in Nuevo Leon; 88 in Tamaulipas; 3 in Coahuila; 0 in Chihuahua; and 0 in Baja California (http://www.dgepi.salud.gob.mx/boletin/2004/sem52). Of all Mexican states, Baja California and Sonora adjacent to the US border have the lowest incidence of flaviviral infections. Human flavivirus serosurveys should be conducted in northern Mexico to further evaluate the possibility of cross-protective flavivirus immunity. Newer approaches to detect and identify flaviviral disease are also needed in Mexico to more accurately assess the impact of WNV.
